# Are fearful boys at higher risk for anxiety? Person-centered profiles of toddler fearful behavior predict anxious behaviors at age 6

**DOI:** 10.3389/fpsyg.2022.911913

**Published:** 2022-08-08

**Authors:** Anna M. Zhou, Austen Trainer, Alicia Vallorani, Xiaoxue Fu, Kristin A. Buss

**Affiliations:** ^1^Department of Psychology, The Pennsylvania State University, University Park, PA, United States; ^2^Department of Psychology, University of South Carolina, Columbia, MO, United States

**Keywords:** temperament, fear, anxiety, early childhood, latent profile analysis

## Abstract

Dysregulated fear (DF), the presence of fearful behaviors in both low-threat and high-threat contexts, is associated with child anxiety symptoms during early childhood (e.g., Buss et al., 2013). However, not all children with DF go on to develop an anxiety disorder (Buss and McDoniel, 2016). This study leveraged the data from two longitudinal cohorts (*N* = 261) to (1) use person-centered methods to identify profiles of fearful temperament, (2) replicate the findings linking DF to anxiety behaviors in kindergarten, (3) test if child sex moderates associations between DF and anxiety behaviors, and (4) examine the consistency of findings across multiple informants of child anxiety behaviors. We identified a normative fear profile (low fear in low-threat contexts; high fear in high-threat contexts), a low fear profile (low fear across both low- and high-threat contexts) and a DF profile (high fear across both low- and high-threat contexts). Results showed that probability of DF profile membership was significantly associated with child self-reported overanxiousness, but not with parent-reported overanxiousness. Associations between DF profile membership and overanxiousness was moderated by child sex such that these associations were significant for boys only. Additionally, results showed that probability of DF profile membership was associated with both parent-reported social withdrawal and observations of social reticence, but there were no significant associations with child self-report of social withdrawal. Results highlight the importance of considering person-centered profiles of fearful temperament across different emotion-eliciting contexts, and the importance of using multiple informants to understand associations with temperamental risk for child anxiety.

## Introduction

Approximately, 10% of preschoolers in the United States meet diagnostic criteria for a DSM anxiety disorder (Egger and Angold, 2006). Anxiety disorders during childhood significantly impact daily functioning. Children with anxiety symptoms tend to avoid social interactions with peers, are perceived as less socially competent, have fewer friendships, and are more likely to be bullied by peers (e.g., Rubin et al., 2009; Huber et al., 2019). Additionally, anxiety symptoms during early childhood are also associated with both severity and chronicity of later anxiety disorders (Pine et al., 1998; Bittner et al., 2007). The early identification of which children are at higher risk for anxiety symptom development may be informative for intervention and prevention of the development of maladaptive socioemotional outcomes. Fearful temperament during toddlerhood is associated with increased risk for later anxiety symptoms during childhood and early adolescence (Degnan and Fox, 2007). However, not all fearful children develop anxiety symptoms or disorders (Degnan and Fox, 2007; Buss and McDoniel, 2016). This highlights that researchers should consider alternative ways to characterize fearful temperament as a marker of anxiety risk and examine how fearful temperament may interact with other factors to better identify which children are at the greatest risk. This study uses person-centered methods to examine how temperamental fear across contexts of varying threat levels may interact with child sex as a predictor of later childhood anxiety outcomes.

### Fear across varying threat contexts as a marker of risk

There is evidence for the normative development of fear from infancy to early childhood (Beesdo et al., 2009; Muris and Field, 2011). For example, fears such as fear of heights and fear of strangers are considered universal and normative during infancy, while fears of animals emerge in toddlerhood (Beesdo et al., 2009; LoBue et al., 2019). Children may express different levels of fears as part of typical development, which differs from children who exhibit persistent or extensive degrees of anxiety and avoidance that are associated with subjective levels of distress and impairment (Beesdo et al., 2009). Additionally, there are individual differences and variability in fearful behaviors, namely temperament, that may be predictive of developmental outcomes such as anxiety symptoms (LoBue et al., 2019).

Temperament refers to biologically based, early emerging individual differences in emotion and its regulation (Shiner et al., 2012). Individual differences in fearful temperament are often considered an early risk factor for the development of anxiety (Klein et al., 2012; Nigg, 2006; Pérez-Edgar and Fox, 2005). Klein et al. (2012) review the multiple perspectives and models in which researchers have considered the relations between temperament and psychopathology, one of which is the predisposition model that posits (1) there are distinct etiological bases to temperament and psychopathology, and (2) there is a complex interplay among multiple risk factors (including temperament) that lead to the development of psychopathology. In this paper, we consider associations between fearful temperament and anxiety symptoms using the predisposition model.

Fearful temperament is a multifaceted construct that includes motivational/affective, behavioral, and physiological aspects of fear (Rothbart and Bates, 2006). One subtype of fearful temperament is behavioral inhibition (BI), which is the tendency to withdraw from and/or exhibit negative affect in response to novelty (Garcia Coll et al., 1984; Kagan et al., 1984; Fox et al., 2005). Both fearful temperament as well as BI have been associated with anxiety symptoms, although not all fearful or behaviorally inhibited children go on to develop an anxiety disorder (e.g., Buss and McDoniel, 2016). While there are some who suggest extreme fearful temperament (i.e., BI) is a prodromal level of anxiety manifesting earlier in development, our view is shaped by Pérez-Edgar and Guyer (2014) who explain and review literature supporting the differences in the two constructs. Consistent with the predisposition model, fearful temperament is distinct from anxiety but is indicative of greater risk for anxiety development. Thus, the focus of the current study is expanding our understanding of which fearful children will develop anxiety symptoms.

Traditional observational approaches to fearful temperament average fearful behavior across highly novel and threatening situations, and do not consider differences in fearful behavior across different tasks of varying threat levels (e.g., Goldsmith and Campos, 1990; Garcia Coll et al., 1984; Talge et al., 2008). Additionally, there is an assumption that fearful behaviors are maladaptive without consideration of the context. Fear is adaptive and normative in contexts where there is threat present. However, when fear is extremely intense and present during situations that are not threatening, fear may be maladaptive and contribute to the development of anxiety (Buss et al., 2004; Goldsmith and Davidson, 2004). Thus, it is important to consider situational context when characterizing fearful temperament as a risk marker for anxiety symptom development.

A previous work identified a type of temperamental fear, called “dysregulated fear (DF),” which is characterized by displays of high-fear behaviors in both low and high-threat contexts (Buss, 2011). Most toddlers in the initial study exhibited what was deemed “normative fear,” a pattern of low levels of fear in low-threat situations, and higher levels of fear behavior in high-threat situations. However, an additional group of toddlers were identified as dysregulated in their pattern of fear as follows: Toddlers who exhibit high levels of fear across all levels of threat, most notable in low-threat tasks. Toddlers exhibiting DF may be experiencing difficulty in appropriately regulating across changing contexts, potentially placing them at higher risk for developing anxiety (Buss and McDoniel, 2016). While DF and BI are conceptually similar, empirical work demonstrates distinctions between a DF profile and a BI profile during toddlerhood (Buss et al., 2004; Buss, 2011) whereby only about one third of toddlers observed would be classified as both BI and DF. One key distinction is how DF reflects a type of high sensitivity to novel stimuli relative to the eliciting contexts vs. just extreme fear averaged across contexts.

The hypothesis that toddler DF is associated with later anxiety symptoms has been supported across two samples wherein DF at age 2 was associated with increased risk of social withdrawal and social anxiety symptom development across early childhood (Buss, 2011; Buss et al., 2013, 2018, 2021a). Specifically, DF during toddlerhood predicted higher ratings of social withdrawal by parents at ages 3, 4, and 5, as well as the ratings of social withdrawal by teachers at age 5 (Buss, 2011). These associations were also replicated when examining associations between DF and social anxiety symptoms reported through clinical interviews with parents at age 5 (Buss et al., 2013). Additionally, toddler DF was associated with observed social reticence during structured lab tasks with an unfamiliar peer during the early childhood (Buss et al., 2013). A previous work has also demonstrated that DF was associated with parents’ report of social anxiety symptoms during the early childhood above and beyond the associations between BI and anxiety symptoms (Buss, 2011). Taken together, DF has been associated with higher levels of social withdrawal and social anxiety across multiple informants and methods of assessment.

While DF is directly associated with parent and teacher reported social withdrawal and social anxiety, there is emerging evidence that DF may be associated with increased risk for generalized anxiety symptoms (e.g., overanxiousness and worry). Prior studies of fearful temperament and BI have demonstrated associations with the maternal report of generalized anxiety symptoms at age 6 (Hudson et al., 2011). Recently, Buss et al. (2021b) found that DF is indirectly associated with maternal reports of children’s general anxiety symptoms *via* parenting behaviors. However, there were no direct associations between DF and child general anxiety symptoms (Buss et al., 2021b). In summary, there are associations between DF and both general anxiety and social anxiety symptoms, although there may only be direct associations between DF and social anxiety symptoms specifically when considering the reports of parent, teacher, and observer reports children’s anxiety behaviors (Buss, 2011; Buss et al., 2013, 2018, 2021b).

It is also important to consider how DF, or other putative patterns of fear across different emotion-eliciting contexts, is measured. Latent profile analysis (LPA) provides a person-centered approach to characterizing temperament types based on different patterns of fear behaviors across different contexts, which may increase the nuance in the identification of temperamental risk during early childhood. Examining the broader temperament literature, the previous studies using latent profile analyses identified different groups of infants and toddlers with differing patterns of temperamental characteristics using parent reports of toddler temperament (Beekman et al., 2015; Gartstein et al., 2017). Additionally, one study found evidence that the profiles of temperament were associated with psychopathology outcomes during middle childhood and early adolescence (Rettew et al., 2008), demonstrating that the person-centered profiles may increase our ability to identify temperamental risk associated with psychopathology outcomes. However, these studies did not examine specific temperamental traits across varying contexts. While there is growing evidence in the literature identifying a profile of DF when considering fearful behavior across varying threat contexts, it is less clear if there may be other subtypes of fearful temperament or patterns of fearful behavior across varying threat contexts. These person-centered profiles may contribute to our understanding of the development of child anxiety and help better identify which children are at greater risk for anxiety development.

### Child sex as a moderator of fearful temperament and child anxiety

Child sex may be another potential factor that moderates associations between fearful temperament and anxiety outcomes. Findings of sex differences in temperament and anxiety during the early childhood period are often small or non-significant. A meta-analysis on gender differences in temperament in children aged 3–13 years only found a very small gender difference between boys and girls in fear (*d* = −0.12) (Else-Quest et al., 2006). Similarly, research on pre-adolescent anxiety finds little evidence for sex differences in anxiety symptoms (e.g., Roza et al., 2003; Jacques and Mash, 2004; Bosquet and Egeland, 2006) although there are studies that find sex differences during adolescence with girls exhibiting higher levels of anxiety symptoms than boys (e.g., Jacques and Mash, 2004). These findings are not surprising as child sex may not have a direct effect on socioemotional development, but instead may play a moderating role (Crick and Zahn-Waxler, 2003). Turning back to the broader temperament literature, fearful boys may be at higher risk for anxiety-related outcomes compared to girls (Kagan et al., 1998; Henderson et al., 2001). Fearful boys exhibited greater social wariness during early childhood compared to fearful girls (Kagan et al., 1998; Degnan and Fox, 2007). In addition, toddler inhibited temperament was significantly associated with social wariness in boys at age 4, but these associations were not significant for girls (Henderson et al., 2001). Additionally, studies have demonstrated that shy boys may be more sensitive to social feedback (Howarth et al., 2013).

These sex differences may be due to the ways in which parents respond to boys’ and girls’ expressions of the same behaviors and emotions, such as fearful behavior (Mills and Rubin, 1990; Park et al., 1997). Prior studies demonstrate that parents respond to boys’ fearful behaviors with greater concern, intrusiveness and protective parenting compared to girls (e.g., Park et al., 1997; Kiel et al., 2016). Additionally, boys and girls may also elicit different parenting behaviors in order to regulate their fear (Buss et al., 2008). Thus, sex differences in how parents respond to their children, as well as how children may rely on their parents to regulate their fearfulness may contribute to sex differences in the associations between fearful temperament and anxiety outcomes.

However, not all studies find moderation by child sex when examining associations between temperament and anxiety. For example, Skarpness and Carson (1986) found that associations between mom-rated inhibition and teacher-rated withdrawal were the same across both boys and girls. Similarly, Morales et al. (2015) found that toddler fearful temperament was associated with parental reports of internalizing behaviors at age 5, but was not moderated by child sex. Additionally, the previous studies examining associations between DF and anxiety/social withdrawal did not find sex differences in either fearful behavior across tasks at age 2 (Buss, 2011) or in the external observers’ ratings of social interactions with an unfamiliar peer at age 6 (Buss et al., 2013). With the mixed available findings in the literature, it is important to consider child sex as a moderator when examining associations between temperament and anxiety.

### Multiple informants of child anxious behavior

Lastly, one major consideration in characterizing risk for anxiety development is utilizing multiple informants of child anxiety. Parent-reported child anxiety is often utilized through questionnaires or structured clinical interviews. However, using measures such as child self-report and observer ratings of child behavior can be informative for developing a comprehensive understanding of child anxiety symptoms by considering the presence of anxiety symptoms in multiple contexts. One challenge of child self-report has been eliciting reliable self-reports due to developmental factors. However, child self-report measures such as the Berkeley Puppet Interview (BPI; Ablow et al., 1999) were designed with developmental considerations for children aged 4-8 years to report on their symptomatology and distress. Research has demonstrated associations between children’s social reticence and self-perceptions of social competence from age group 4-7 years (Nelson et al., 2005), thus highlighting the importance of considering children’s self-ratings of anxiety symptoms. While there have been low levels of agreement between different informants in past studies (Achenbach et al., 1987; Ablow et al., 1999), a previous study has also demonstrated that young children’s self-report of core anxiety symptoms are associated with parent report when measures are administered concurrently (Luby et al., 2007). In particular, children are able to accurately report on behaviors such as being shy with peers and having bad dreams (Luby et al., 2007).

### The current study

This study had the following four main goals: (1) To use person-centered methods to identify profiles of fearful temperament, (2) to replicate of findings linking DF to anxiety behaviors in kindergarten, (3) to test if child biological sex is a moderator of associations between DF and later anxiety behaviors, and (4) to examine the consistency of findings across multiple informants of child anxiety behaviors. The extant literature provides evidence that variation in fearful behavior across contexts may indicate risk for anxiety development (e.g., Buss, 2011; Buss et al., 2018). However, the previous studies of DF may have been limited by sample size to identify different profiles of fearful behavior across contexts using a person-centered approach. This study combined two samples of children, including the participants from the previous studies (e.g., Buss et al., 2013; 2018) to generate a larger sample size to examine if there may be profiles of fearful behavior across varying threat contexts beyond the normative and DF profiles. To address the first goal, we used LPA to identify different profiles of fearful temperament across six fear-eliciting episodes of varying threat contexts. Only one previous study of DF used LPA in a single sample, which identified the following two patterns of fearful behavior across profiles: A normative fear subtype characterized by low fear in low-threat contexts and high fear in high-threat contexts, and a DF subtype characterized by high fear in both low- and high-threat contexts (Buss, 2011).

As the previous literature demonstrated associations between DF and later anxiety (Buss, 2011; Buss et al., 2013, 2018), we hypothesized that DF will be associated with anxiety symptoms associated with generalized anxiety (or overanxiousness) and social withdrawal. We aimed to replicate these associations with a larger sample size by combining two cohorts to increase statistical power. Additionally, we considered child sex as a moderator of the association between profiles during toddlerhood and anxiety symptoms during early childhood. Through leveraging two cohorts of children, we may have a larger sample size that is able to detect moderation.

Finally, we examined if the associations were robust across child self-report, parental report of child general and social anxiety symptoms, and behavioral coding of social withdrawal when interacting with a novel peer. The previous studies examining associations between DF and child anxiety have not utilized child self-report in the past. Thus, the current study aimed to extend the extant literature by incorporating measures of child self-report of anxiety and examining if associations with parent-report and observed social reticence are consistent with associations with child self-report. We hypothesized that higher likelihood of DF profile membership would be positively associated with anxiety symptoms at age 6, and that these associations would be stronger for boys than for girls. Additionally, we expected to find robust associations across child self-report and parental report of child anxiety symptoms, and behavioral coding of social reticence when interacting with a novel peer. Thus, we hypothesized that the associations between DF and anxiety symptoms would be similar across different informants and when examining behavior.

## Materials and methods

### Participants

The data was obtained from 261 children (54% boys, 46% girls, *M_*age*_* = 24.39 months, SD = 1.4 months) and parents participating in a two-cohort longitudinal study of toddler’s temperament and socioemotional development in age group 2–6 years. The participants for both cohorts were recruited using community-based sampling, such as birth announcements and a database of families interested in participating in research, from small Midwestern and Northeastern cities and their surrounding rural counties (Cohort 1 and Cohort 2, respectively). As part of the larger longitudinal study, toddlers for Cohort 2 were oversampled for anxiety risk and high fear at 18 months (see Buss, 2011 and Buss et al., 2018, for full sampling details).

Of participating families, 84% identified as White, 6% identified as Black, 4% identified as Asian, 2% identified as Hispanic, 1% of families identified as American Indian, and 3% identified as another race/ethnicity or did not respond. A total of 42.5% of participating families reported an income of over $60,000 a year, 33.5% of families reported an income of between $31,000 and $59,000, 12% reported an income of $29,000 or less, and 12% did not respond. 25.3% of mothers reported having completed high school and some college, 28.3% reported having completed their undergraduate degree, 22.7% reported having 1–3 years of graduate education, 9.7% of mothers reported having completed 4+ years of graduate education, and 14% of mothers did not report their education level. A total of 31.6% of fathers reported having completed high school and some college, 27% reported having completed their undergraduate degree, 15.6% reported having 1–3 years of graduate education, 12.2% reported having completed 4+ years of graduate education, 1% reported having not completed high school, and 12.7% of fathers did not report their education level.

### Procedures and measures

#### Age 2 procedures and measures

During the first laboratory visit at age 2, mothers and children participated in a series of six tasks modeled from the toddler version of the Laboratory Temperament Assessment Battery and other fear-eliciting laboratory tasks (Toddler Lab-TAB; Nachmias et al., 1996; Buss and Goldsmith, 2000). Toddlers were introduced to six novel emotion-eliciting stimuli of varying threat levels, including watching a puppet show (low threat), being approached by an unfamiliar adult (medium threat), and being approached by automated toy spider (high threat). Toddlers were allowed to respond naturally (see for full description of tasks Buss, 2011).

Each task began with toddlers seated on their mother’s lap. “Clown” task and “puppet show” were designed to be novel but engaging and non-threatening (i.e., low threat). During the clown task, a female experimenter entered the room dressed as a clown (i.e., multicolored wig, red nose) and invited the child to play with them. During the puppet show, two animal puppets played games with each other and invited the child to play with them. Two tasks were designed around interacting with strangers and considered to be moderate threat. In the “stranger working,” a female stranger would enter the room and pretend to work, not interacting with the child unless initiated by the child. In stranger approach, a male stranger wearing a baseball cap would enter the room, slowly approaching the child, asking the toddler several short questions (e.g., “Are you having fun today?”). The high-threat tasks included Spider and Robot, in which toddlers began seated in their mother’s lap facing a small animatronic object (a large plush spider affixed to an RC car or small anthropomorphic robot, respectively). The Robot sat motionless for a brief period and then moved around on a platform, making sounds, and lighting up. During Spider, after a similar period of inactivity the spider moved slowly toward the child and retreated (this occurred twice).

##### Observed fear during toddlerhood

Children’s observed fear was calculated using second-by-second coding of facial fear, bodily expressions of fear, freezing, and proximity to caregivers during each task. Facial fear was coded using the AFFEX coding system (Izard et al., 1983), while bodily fear was coded as the presence and duration of bodily expressions of fear such as diminished play. Freezing was coded as the child becoming still or rigid in response to a stimulus for durations of 2 s and longer. Proximity to mother was calculated using the duration of time spent in maximum proximity to caregiver (i.e., sitting on mother’s lap, physically touching). Finally, the latency to freeze was measured as the number of seconds between the beginning of the task and the first onset of freezing behaviors. A composite of the duration fear behaviors was calculated (with latency to freezing reverse coded) and transformed into a proportion score (divided by total length of the episode).

Coders were rated at 90% interrater agreement and levels of internal consistency were acceptable for both cohorts (Buss et al., 2008, Buss, 2011). As such, the behaviors were averaged and compared to the total length of each episode to determine the percentage of each episode spent engaging in fearful behavior per episode.

#### Age 6 procedures and measures

Families were invited to participate in a multipart assessment during the fall and spring of children’s kindergarten year. After agreeing to participate, the parents completed a series of at home questionnaires prior to the first of two laboratory visits (typically within one week). During the first laboratory visit, the children participated in a variety of tasks, including the Berkeley Puppet Interview (BPI; Measelle et al., 1998) in which children provided self-reports of their socially inhibited and anxious behaviors. During this task, the parents remained in another room so as not to influence children’s responses; children received a small prize after completing the interview.

During the spring of the kindergarten year, parents who had expressed interest in participating in the study completed another series of questionnaires about children’s socioemotional adjustment. Families returned to the lab for their second visit, in which children participated in a laboratory peer-visit. During this task, groups of 3–4 years of age, unfamiliar, same-sex children engaged in a 15-min free play episode as part of the activities under the Play Observation Study (Rubin, 1989). Children were provided a variety of activities and instructed to play “however you like.”

##### Parent report of overanxiousness and social withdrawal

Parent reports of children’s overanxiousness and social withdrawal were assessed using parent versions of the MacArthur Health and Behavior Questionnaire (HBQ; Armstrong et al., 2003). The parents were asked to report how accurately a variety of descriptions and behaviors represented the child on a scale from 0 = never/not true, 1 = sometimes/somewhat true, or 2 = often or very true. The overanxiousness subscale was composed of 12 items (i.e., “Has nightmares”, or “Is Self-Conscious or easily embarrassed”), which were averaged to create one composite overaxiousness score (*M* = 0.39, *SD* = 0.24). Responses for parents and teachers were found to be reliable across both cohorts (Cohort 1 α = 0.70, Cohort 2 α = 0.66). Social withdrawal (*M* = 0.44, *SD* = 0.24) was measured as the mean of the 6-item Asocial with Peers subscale (i.e., “Is a solitary child,” “prefers to play alone”) and the 3-item Social Inhibition subscale (i.e., “Shy with other children” and “Is afraid of strangers”). Responses for parents and teachers were found to be reliable across both cohorts (Cohort 1 Parent α = 0.77, Cohort 2 Parent α = 0.76).

##### Child self-report of overanxiousness and social withdrawal

Children’s self-report of overanxiousness and social withdrawal were assessed using children’s video recorded responses to the BPI. During the BPI, each of the hand puppets offered opposing statements describing themselves such as “I have lots of friends at school” and “I don’t have lots of friends at school,” and then asked the child “What about you?” Children’s responses were coded by trained coders on a 7-point Likert scale in which very positive self-reports (e.g., “I’m friends with everyone at school”) were coded at one endpoint (1), and very negative self-reports (e.g., “I have no friends at school”) were coded at the other endpoint (7). Interrater agreement (Cohort 1 = 96%, Cohort 2 = 93%) and reliability (Cohort 1 κ = 0.90, Cohort 2 κ = 0.83) were acceptable for both cohorts.

The overanxiousness subscale was composed of 6 items (i.e., “I have/don’t have lots of bad dreams” and “I worry/don’t worry a lot”), which were averaged to create one composite overanxiousness score (*M* = 5.07, *SD* = 0.90; Cohort 1 α = 0.52, Cohort 2 α = 0.50) As with the HBQ, social withdrawal (*M* = 4.88, *SD* = 0.88; Cohort 1 α = 0.70; Cohort 2 α = 0.75) was assessed using the mean of the Asocial with Peers subscale (five items, i.e., “I’d rather play games by myself/with lots of kids”) and the Social Inhibition subscale (six items, i.e., “I worry/don’t worry if other kids will like me”). Internal consistency for scales on the BPI are often lower for children recruited from the community compared to a clinic-referred sample as items with low base rates in a non-clinical samples can contribute to decreased internal consistency (Ablow et al., 1999).

##### Observed social reticence

Children’s social reticence was assessed through observational coding of children’s behaviors during the peer-visit task using the Play Observations Scale (POS; Rubin, 1989). Coders scored for a wide range of play and non-play behaviors in 10-s epochs with only one play behavior coded for each epoch. In line with Rubin, 1989, when multiple play behaviors occurred within a given epoch the behavior observed for the majority of the epoch was coded as predominant. Among these behaviors, unoccupied behavior was coded when children were staring blankly or wandering without purpose. Onlooking behaviors included watching children from a distance (e.g., further than three feet) without attempting to join the activity. As per a later work by Rubin et al. (e.g., Coplan et al., 1994; Rubin et al., 2002), these behaviors were collapsed into a single social reticence code containing any instance of unoccupied or onlooking behaviors. The proportion of time children were socially reticent (number of epochs in which social reticence was predominant divided by the total number of epochs) were computed and used for analyses. Interrater agreement (Cohort 1 = 93%, Cohort 2 = 94%) and reliability (Cohort 1 κ = 0.61, Cohort 2 κ = 0.85) were acceptable for both cohorts.

### Data analytic plan

#### Missing data

There were data missing in this study (see Table 1 for the number of the participants who provided data for each variable of interest). There were 22 patterns of missing data, and Little’s MCAR test indicated that data was missing completely at random, χ^2^ = 229, *p* = 0.06. However, there were more missing data from Cohort 1 participants than Cohort 2 for the age 6 anxiety measures as well as sampling differences between the two cohorts, and so cohort was included as a covariate in all analyses with anxiety measures. Full information maximum likelihood (FIML) estimator in multiple regression models with missing data have shown to produce less biased parameter estimates, especially compared to listwise deletion, pairwise deletion and mean imputation (Enders, 2001). As such, all multiple regressions in this study were conducted using FIML.

**TABLE 1:** Means and standard deviations of key variables of interest by child gender.

	Full sample			Boys			Girls		
			
Variable	*M*	*SD*	*n*	*M*	*SD*	*n*	*M*	*SD*	*n*
**Proportion of Fear**									
Clown	24.70	22.64	261	24.31	23.41	141	25.17	21.79	118
Puppet show	30.60	22.23	259	30.25	23.66	141	31.02	22.80	118
Stranger working	23.17	17.09	259	22.81	18.33	142	23.61	15.51	115
Stranger approach	24.67	19.97	254	23.72	20.63	138	25.79	19.17	116
Robot	55.53	28.09	255	54.96	23.75	138	59.73	25.50	117
Spider	54.24	22.41	256	52.83	23.70	140	55.95	20.72	116
**Anxiety behaviors at age 6**									
BPI overanxiousness	5.07	0.90	128	5.04	0.91	69	5.11	0.90	59
BPI social withdrawal	4.88	0.88	128	4.81	0.89	68	4.94	0.90	59
Health and Behavior Questionnaire (HBQ) overanxiousness	0.39	0.24	157	0.39	0.25	84	0.38	0.22	73
HBQ social withdrawal	0.44	0.30	157	0.45	0.32	84	0.42	0.28	73
POS reticence	0.10	0.14	141	0.11	0.17	76	0.09	0.09	65

#### Latent profile analyses

To identify the latent groups of the participants based on observed patterns of behaviors across the six fear-eliciting episodes, LPA was employed to estimate the profiles and the probability of profile membership for each individual within the same model (Mplus version 5.1; Muthén and Muthén, 2007). The latent profile models specified with two, three, and four latent classes were evaluated. The best fitting model was selected based on the fit statistics (i.e., BIC and AIC), entropy, and bootstrapping likelihood ratio tests, as well as theoretical considerations.

#### Regression analyses

Multiple regressions were conducted using the *lavaan* package (Rosseel, 2012) in R (R Core Team, 2020). We used the probability of profile membership in the DF group to characterize DF using the full sample (*N* = 261). We ran five regression models to examine if child sex moderated the following associations between DF and different child anxiety outcomes: (1) Child self-reported overanxiousness, (2) child self-reported social withdrawal, (3) parent-reported overanxiousness, (4) parent-reported social withdrawal, and (5) observer coding of social reticence during the play task. Cohort was also included as a covariate in all models. When significant interactions were detected, simple slope tests (at 1 SD above and below the mean, and at the mean) were embedded within the model to help interpret the interaction terms.

## Results

### Descriptive statistics

Table 1 contains the means, standard deviations, and sample sizes of key variables of interest by child sex.

Table 2 contains bivariate correlations of all key variables of interest. Child report of overanxiousness was significantly, positively associated with child report of social withdrawal (*r* = 0.24), and parent report of overanxiousness was positively correlated with parent report of social withdrawal (*r* = 0.43). Across informants, child and parent reports of anxiety were not correlated with each other, but there was a significant, positive correlation between parent report of social withdrawal and observed reticence during the peer interaction task (*r* = 0.24).

**TABLE 2:** Bivariate correlations of key variables of interest.

Variable	1	2	3	4	5	6	7	8	9	10	11
**Proportion of fear**											
(1) Clown	1.00										
(2) Puppet Show	0.43*	1.00									
(3) Stranger Working	0.36*	0.34*	1.00								
(4) Stranger Approach	0.33*	0.24*	0.26*	1.00							
(5) Robot	0.19*	0.21*	0.17*	0.13*	1.00						
(6) Spider	0.26*	0.15*	0.06	0.06	0.35	1.00					
**Anxiety behaviors at age 6**											
(7) BPI overanxiousness	0.14	0.26*	0.08	–0.05	0.14	0.09	1.00				
(8) BPI Social withdrawal	0.17	0.19*	0.25*	0.04	0.17*	0.17*	0.24*	1.00			
(9) HBQ overanxiousness	0.15	0.06	–0.09	–0.02	–0.06	–0.06	–0.05	–0.05	1.00		
(10) HBQ social withdrawal	0.17*	0.27*	0.11	–0.01	0.01	0.01	0.12	0.02	0.43*	1.00	
(11) POS reticence	0.21*	0.13	0.05	0.16	0.05	0.05	0.09	–0.02	–0.03	0.24*	1.00

*p < 0.05.

### Latent profile analysis

For the two-profile solution, the entropy was greater than 0.80, indicating this solution had adequate precision of individual profile membership (Celeux and Soromenho, 1996). The significant VLMR-LRTs for the two- and three-profile models suggested further consideration of these models. The BIC value was smallest for the three-profile model, and the entropy value of the three-profile model is close to 0.80. Hence, we selected the three-profile model as the best-fitting model due to both fit indices as well as theoretical considerations (Table 3).

**TABLE 3:** Fit for latent profile models of age 2 fear behavior.

	1-Profile	2-Profile	3-Profile	4-Profile	5-Profile	6-Profile
**Information criteria**						
AIC	13907.01	13713.87	13675.56	13658.32	13649.93	13628.06
BIC	13949.78	13781.60	13768.24	13775.95	13792.51	13795.60
Adj. BIC	13911.75	13721.36	13685.81	13671.32	13554.69	13646.59
Log likelihood	–6941.504	–6837.934	–6811.780	–6796.158	–6784.963	–6767.032
Model convergence	Yes	Yes	Yes	Yes	Yes	Yes
Entropy	–	0.873	0.772	0.833	0.776	0.836
**Relative fit tests**						
VLMR	–	*p* = 0.0000	*p* = 0.0108	*p* = 0.1828	*p* = 0.8315	*p* = 0.2986
LMR Adj.	–	*p* = 0.0000	*p* = 0.0 0122	*p* = 0.1908	*p* = 0.8347	*p* = 0.3057
Bootstrapped LRT	–	*p* = 0.0000	*p* = 0.0000	*p* = 0.0000	*p* = 0.0984	*p* = 0.0000

**FIGURE 1:**
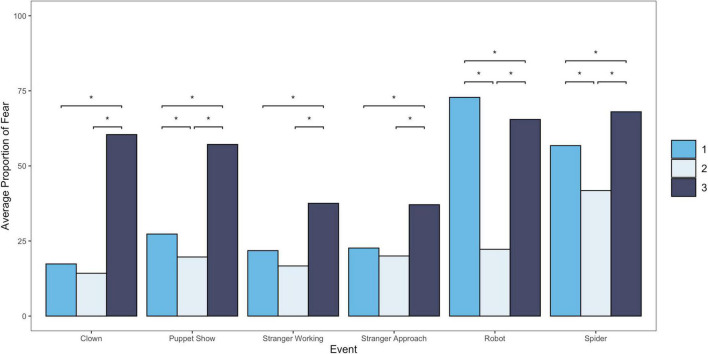
Bar chart showing average proportion of fear for each event by profile, with significant differences between profiles indicated. *p < 0.05.

Through visual inspection and statistical tests, all three profiles of fear across threat contexts were significantly different between groups across tasks. Average fear across each episode by profile can be found in Table 4 and Figure 1. One-way analyses of variance (ANOVAs) indicated that there were statistically significant differences between groups on fear across all tasks [clown task: *F*(2, 250) = 184.70, *p* < 0.01; Puppet Show: *F*(2, 250) = 62.08, *p* < 0.01; stranger working: *F*(2, 248) = 28.63, *p* < 0.01; stranger approach: *F*(2, 245) = 12.36, *p* < 0.01; Robot: *F*(2, 246) = 233.70; *p* < 0.01; Spider *F*(2, 247) = 27.16, *p* < 0.01]. Tukey *post hoc* tests were conducted to examine differences between specific profiles for each task. Profile 1 (*N* = 127) was labeled as “normative fear,” as children exhibited low levels of fearful behaviors in low-threat tasks but exhibited higher levels of fear during high-threat tasks. Profile 2 (*N* = 84) was characterized by low fear across all tasks, thus labeled “low fear.” Lastly, children in Profile 3 (*N* = 50) exhibited patterns of higher levels of fearful behavior across both high- and low-threat tasks and was thus labeled “dysregulated fear.”

**TABLE 4:** Means and standard deviations for fear during each Lab-TAB event by profile.

Profile	Clown *M* (*SD*)	Puppet show *M* (*SD*)	Stranger working *M* (*SD*)	Stranger approach *M* (*SD*)	Robot *M* (*SD*)	Spider *M* (*SD*)
(1) Normative fear	17.61 (12.58)	27.18 (19.83)	21.77 (14.74)	23.15 (18.85)	73.20 (14.98)	57.06 (20.87)
(2) Low fear	14.31 (16.22)	19.57 (15.91)	16.65 (14.15)	20.02 (20.14)	22.46 (15.43)	41.66 (22.14)
(3) Dysregulated fear (DF)	60.57 (15.57)	56.94 (21.59)	37.55 (18.33)	36.65 (17.03)	65.92 (21.88)	68.38 (16.36)

### Does profile membership interact with child sex to predict anxiety symptoms at age 6?

#### Overanxiousness

##### Associations with child self-report

**FIGURE 2:**
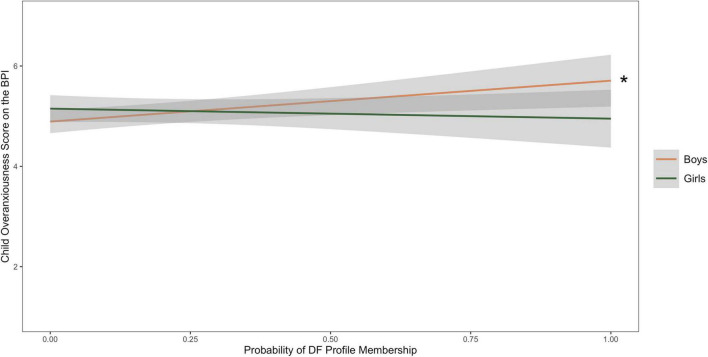
Line graph depicting associations between the probability of dysregulated fear (DF) profile membership and child self-report of overanxiousness on the BPI, with child sex as a moderator. The symbol * indicates a significant slope (*p* < 0.05).

Table 5 contains the results of the multiple regression with probability of membership in the DF profile, child sex, their interaction as predictors of child self-reported overanxiousness, with cohort as a covariate (*R*^2^ = 0.08, *f*^2^ = 0.09). The probability of DF profile was significantly associated with child self-report of overanxiousness, β = 1.90, *p* < 0.01, while there were no main effects of child sex. The interaction between probability of profile membership and child sex was significantly associated with overanxiousness symptoms. Simple slopes testing demonstrated that probability of DF profile is positively associated with overanxiousness for boys (β = 0.96, *p* < 0.01), while probability of DF profile was not significantly associated with overanxiousness for girls (β = 0.00 *p* > 0.05). Figure 2 depicts the interaction between probability of DF profile membership and child sex to predict child self-reported overanxiousness.

**TABLE 5:** Summary of multiple regression of membership in dysregulated fear (DF) profile, child sex, and their interactions on child self-report of overanxiousness on the BPI.

Predictors	Estimate	SE	95% CI		*p*
				
			Lower	Upper	
Cohort	–0.227	0.164	–0.548	0.094	0.165
Probability of DF membership	1.837	0.656	0.003	0.307	0.005
Child Sex	0.078	0.154	–0.223	0.379	0.611
Probability of DF × Sex	–0.956	0.426	–1.792	-0.121	0.025

##### Associations with maternal report

Multiple regressions with probability of profile membership, child sex, and their interaction as predictors of maternal report of child overanxiousness on the HBQ at age 6, with cohort as a covariate were not significant (*R*^2^ = 0.02, *f*^2^ = 0.02).

#### Social withdrawal

##### Associations with child self-report

Multiple regression with probability of DF membership, sex, and their interaction were not significantly associated with child self-report of social withdrawal on the BPI after controlling for cohort (*R*^2^ = 0.08, *f*^2^ = 0.09).

##### Associations with maternal report

**FIGURE 3:**
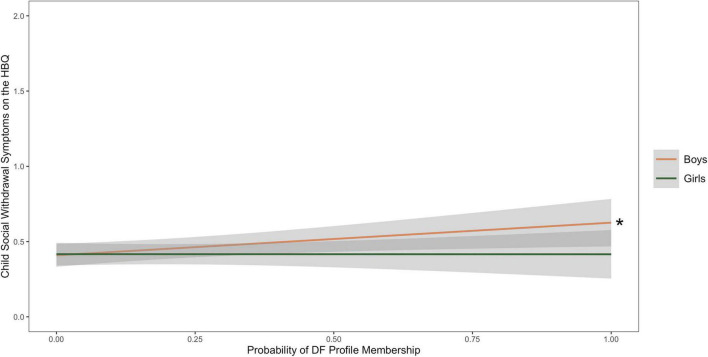
Line graph depicting associations between the probability of dysregulated fear (DF) profile membership and parental report of social withdrawal symptoms on the Health and Behavior Questionnaire (HBQ), with child sex as a moderator. Other multiple regressions with probability of profile membership, child sex, and their interaction as predictors of maternal report of child social withdrawal on the Health and Behavior Questionnaire (HBQ), with cohort as a covariate were not significant (*R*^2^ = 0.01, *f*^2^ = 0.01). The symbol * indicates a significant slope (*p* < 0.05).

A multiple regression with the probability of DF membership, sex, and their interaction were significantly associated with maternal report of social withdrawal on the HBQ at age 6 after controlling for cohort (*R*^2^ = 0.04, *f*^2^ = 0.04). Table 6 contains the results of the multiple regression with probability of membership in the DF profile, child sex, their interaction as predictors of child-reported social withdrawal on the BPI, with cohort as a covariate. The probability of membership in the DF profile was significantly associated with parent report of social withdrawal, β = 0.43, *p* = 0.03, while there were no main effects of child sex. The interaction between probability of profile membership and child sex was trending toward significance. Figure 3 depicts the interaction between probability of DF profile membership and child sex to predict maternal report of social withdrawal.

**TABLE 6:** Summary of multiple regression of membership in dysregulated fear (DF) profile, child sex, and their interactions on maternal report of social withdrawal on the Health and Behavior Questionnaire (HBQ) at child age 6.

Predictors	Estimate	SE	95% CI		*p*
				
			Lower	Upper	
Cohort	0.003	0.049	–0.093	0.099	0.947
Probability of DF membership	0.434	0.196	0.046	0.386	0.026
Child Sex	–0.035	0.047	–0.128	0.058	0.462
Probability of DF × Sex	–0.218	0.127	–0.468	0.031	0.086

##### Associations with observed reticence during a peer task

**FIGURE 4:**
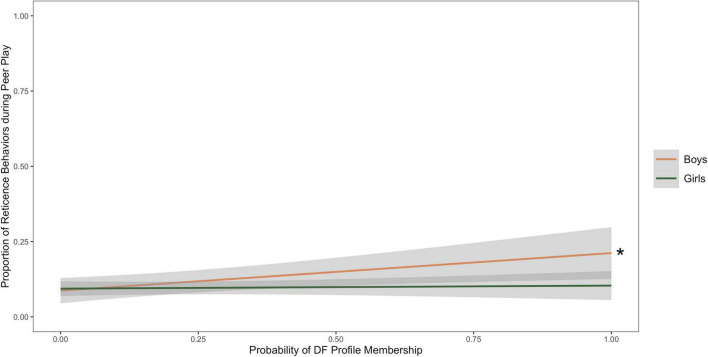
Line graph depicting associations between the probability of dysregulated fear (DF) profile membership and proportion of reticence behaviors during a peer play task, with child sex as a moderator. The symbol * indicates a significant slope (*p* < 0.05).

A multiple regression with the probability of DF membership, child sex, and their interactions on reticence during a peer interaction task with cohort as a covariate (*R*^2^ = 0.08, *f*^2^ = 0.09). Table 7 contains the results, showing a main effect of probability of DF membership was positively associated with reticence (β = 0.13 *p* < 0.01). Additionally, the interaction between probability of DF membership and child sex was trending toward significance (β = −0.10 *p* = 0.06), with simple slopes demonstrating that probability of DF membership is positively associated with reticence for boys (β = 0.11, *p* = 0.01), while probability of DF membership was not significantly associated with reticence for girls (β = 0.01 *p* > 0.05). Figure 4 depicts the interaction between the probability of DF profile membership and child sex to predict external observers’ scores of social reticence.

**TABLE 7:** Summary of multiple regression of membership in dysregulated fear (DF) profile, child sex, and their interactions on behavioral coding of reticence during a peer play task.

Predictors	Estimate	SE	95% CI		*p*
				
			Lower	Upper	
Cohort	–0.029	0.021	–0.076	0.015	0.168
Probability of DF membership	0.126	0.037	0.057	0.215	0.001
Child Sex	–0.013	0.020	–0.057	0.029	0.515
Probability of dysregulated fear DF × Sex	–0.102	0.053	–0.223	0.002	0.055

## Discussion

The current study extends the findings of previous studies on fearful temperament as a marker of anxiety risk by using person-centered methods to characterize fearful temperament across six tasks of varying threat. The results were largely consistent with our hypotheses that DF was associated with features of social anxiety. Additionally, we found evidence that child sex may moderate associations between DF and child anxiety symptoms, such that toddler boys exhibiting DF may be at higher risk for developing later anxiety symptoms during early childhood. Additionally, we found the differential associations between fearful temperament and child anxiety symptoms during the early childhood period depending on the reporter of anxiety symptoms, highlighting the importance of utilizing a multi-method and multi-reporter approach.

### Three-profile solution for fearful temperament across contexts

The uniqueness of our person-centered approach enabled us to characterize fearful temperament by examining fearful behaviors across contexts, instead of considering fearful temperament as an average of fearful behavior across tasks. From the analyses conducted, we find evidence for three different profiles of temperamental fear across threat levels. Consistent with the findings from Buss (2011), we identified patterns of fearful behavior across contexts that were normative (low fear in low-threat contexts, and high fear in high-threat contexts) as well as dysregulated (high fear in both low- and high-threat contexts). We expected most children to express lower levels of fearful behavior in low-threat contexts, and higher levels of fearful behavior in high-threat contexts, a pattern that is consistent with adaptive fear and stress responses in humans. Additionally, consistent with the previous studies, we identified a group of children who did not appear to regulate their emotions across different contexts and displayed high levels of fear across both low- and high-threat contexts. It is important to note that children in the DF profile do not always exhibit more fear that other children, especially other types of fearful children. Because fear can be adaptive in high-threat contexts we have reported elsewhere that DF children are indistinguishable if only observed in high-threat contexts (Buss, 2011). Instead, DF children exhibit fearful behaviors even in low-threat contexts, which sets them apart from the normative fear profile and likely indicates their lack of ability to regulate their fear across different contexts.

Additionally, through increasing the sample size by leveraging data from two cohorts, we were able to identify a third profile of low fear (low fear in both low- and high-threat contexts). It is possible that children in the low fear group may be exuberant or surgent, and exhibit higher levels of approach (Putnam and Stifter, 2005). Exuberant and surgent children have been found to display lower levels of fearful behavior even in the context of higher levels of threat (Fox et al., 2001). Additional work suggests these children may be higher in approach toward novelty and may also be indicative of poor (or inappropriate) emotional reactivity to high-threat contexts. More work is needed to replicate our findings as there has not been work done on children expressing low fear (and possibly high approach) across varying contexts of low and high threat. It could be that this pattern of low fear across low- and high-threat contexts may be one way to characterize exuberant temperament, and may play a role in identifying which of these children may be at risk for developing externalizing symptoms (Degnan et al., 2011).

### Fearful boys may be at greater risk for anxiety

Our findings using person-centered methods are consistent with the previous studies that show DF as a marker of anxiety risk. Additionally, our findings suggest that DF may be a risk factor for anxiety development specifically for boys during the early childhood period. We found that DF was positively associated with child self-reported general anxiety symptoms in boys. Additionally, the interaction between DF and child sex was approaching significant in the models examining parent-reported social withdrawal and observed social reticence. While the results should be interpreted with caution given that they were not significant, the findings provide preliminary evidence that DF may be positively associated with both parent-reported social withdrawal and observed reticence in boys, but not in girls. Taken together, we find evidence that child sex may moderate some associations between DF and anxiety, and that in particular, fearful boys might be at higher risk of anxiety development during this early childhood period.

Some of the findings in this study are consistent with the previous research demonstrating associations between inhibited temperament and social wariness in young boys but not in girls (Henderson et al., 2001). One mechanism through which sex differences may emerge could be through parenting behaviors and socialization of emotion. There is growing evidence that fearful temperament is associated with child anxiety through maternal protective behavior (Kiel et al., 2016; Buss et al., 2021b). Additionally, Kiel et al. (2016) demonstrate that child sex interacts with DF and maternal accuracy in relation to protective parenting behaviors, and that this may be a pathway by which DF is associated with later anxiety. Parents may respond to their children’s fearfulness differently based on child sex, as some evidence suggests parents respond to boys’ fearful behaviors with greater concern, intrusiveness and protective parenting compared to girls (e.g., Park et al., 1997; Kiel et al., 2016). Additionally, the previous studies also show that boys and girls may differ in seeking out their parents for help with regulating distress, and there were only associations between distress during fear-eliciting tasks with contact-seeking for boys, not girls (Buss et al., 2008). Future work should examine the role that protective parenting, or parental socialization of emotion may differ for fearful boys vs. fearful girls, and how that may be a pathway by which fearful temperament is associated with later anxiety outcomes.

### Multiple informants of child anxiety

Lastly, one of the strengths of the current study is the use of multiple informants to assess child anxiety and social withdrawal. Contrary to our hypotheses, we did not find consistent robust associations between fearful temperament and anxiety outcomes for different measures of anxiety. We did not find significant associations between DF and child self-report of social withdrawal, and we did not find associations between DF and maternal report of child overanxiousness. Of note, child self-report measures of overanxiousness and social withdrawal were not significantly correlated with parent-reported overanxiousness and social withdrawal as well as observed social reticence. A previous research in an adolescent sample found that child- and parent-reported anxiety are differentially associated with anxious behaviors in different contexts (Bowers et al., 2020). As such, parent report of social withdrawal is more strongly associated with observational coding of reticence during an unfamiliar play task likely because parents have had the opportunity to observe their children in these situations. On the other hand, child self-report of social withdrawal not being associated with observed reticence at this early developmental period may not be that surprising. One possibility to explain the consistency between parent and external observer reports of social withdrawal is that both measures may be based more on observable anxiety behaviors of the child, whereas child self-report may reflect elements of children’s understanding of emotion and cognitive processes. There is evidence that children exhibit self-appraisal biases in social situations (Lau et al., 2022), which could lead to disparities between child self-report with observer (including parental) reports. Additionally, research on children’s social withdrawal demonstrate that there are shy children who are motivated to seek out peers and social interactions, but are too shy to do so (Coplan et al., 2004). This highlights the importance of considering multiple informants, as they may capture different processes underlying social withdrawal and reticence in children.

At the same time, with the age of the sample, findings with child self-report should be viewed with caution. While the BPI has been established as a valid instrument to collect child self-report of clinical symptoms when children are between 4 and 8 years of age, it is important to note that reliabilities for child self-report of overanxiousness was relatively low in this sample (α = 0.51). Ablow et al. (1999) found that internal consistency for scales on the BPI were often lower for children recruited from the community compared to a clinic-referred sample. It is possible that for items with low base rates in a non-clinical sample can contribute to decreased internal consistency, and the samples in the current study were not clinically referred. One other possibility is that some research demonstrates children may endorse items regardless of their content and acquiesce more during interviews due to developing socioemotional competencies and understanding of emotion, leading to more inconsistencies across scales (Soto et al., 2008; Denham et al., 2009). Future studies should extend the current findings to examine if there may be differences in ways children are interpreting their own emotions and feelings of anxiety compared to how parents and external observers are observing their anxiety behaviors across the later developmental periods.

### Limitations

The current findings should be interpreted within the context of design limitations. First, the sample is majority white and relatively low risk, although we oversampled for fearful behaviors during toddlerhood for Cohort 2. Future studies should examine whether these findings may extend to other populations. Although mean anxiety levels across the sample are consistent with community samples (Ablow et al., 1999), studies have demonstrated poorer internal consistency within community samples compared to clinical samples. This work should be replicated in higher risk samples with higher endorsement of anxiety symptoms to assess if these associations between DF and anxiety are still present, as there may be differences in fearful temperament profiles for children developing in different contexts. Additionally, as factors such as parenting or parental socialization of emotion were not examined, our understanding of why fearful boys may be at higher risk for anxiety development is limited. Future research should aim to explore the mechanisms that may place boys at higher risk for anxiety development if they exhibit profiles of DF, such as observing parenting behavior in response to children’s fearful behaviors across contexts or considering parental socialization of emotion as a pathway. Future studies should also consider the differential roles that mothers and fathers may play, as the previous studies show that fathers may respond differently to children’s negative emotionality compared to mothers (Engle and McElwain, 2011). Lastly, while this study is a longitudinal study, repeated measures of child anxiety symptoms were not assessed. Examining change in fearful temperament and/or child anxiety symptoms can further our understanding of how risk factors influence the developmental trajectories of anxiety development across time. Although a previous work demonstrates that girls exhibit higher levels during adolescence (Letcher et al., 2012), future work should examine if fearful temperament is associated with sex differences in trajectories of anxiety development from early childhood through adolescence.

## Conclusion

This study provides evidence that it is important to consider the emotion-eliciting contexts in which fearful behavior occurs to characterize fearful temperament. Consistent with the previous literature, children who display elevated fear across contexts of varying threat are at greater risk for anxiety development. This study extends the extant literature by leveraging two cohorts to increase power to detect multiple patterns of fearful behavior across context, identifying a low fear profile in addition to replicating patterns of normative fear and DF. Additionally, this study contributes to the literature on DF as a marker of risk for anxiety by findings showing higher risk for DF boys, and by utilizing child self-report outcomes. Our study highlights the importance of (1) considering fearful temperament as characterized by behavior across different contexts and (2) using multiple informants to assess anxious behaviors during childhood. Through extending our knowledge of how to characterize risk for anxiety development across different contexts and using multiple measures, we can better understand how to best identify children at the greatest risk for anxiety development.

## Data availability statement

The data analyzed in this study is subject to the following licenses/restrictions. The data are not publicly available due to privacy or ethical restrictions. Requests to access these datasets should be directed to KB, kab37@psu.edu.

## Ethics statement

The studies involving human participants were reviewed and approved by University of Missouri Human Subjects Research Office and The Penn State Office of Research Protections. Written informed consent to participate in this study was provided by the participants’ legal guardian/next of kin.

## Author contributions

KB and AZ conceived and designed the study. AZ, AT, AV, and XF contributed to the statistical analyses presented in the manuscript. AZ wrote the first draft of the manuscript. AT wrote sections of the manuscript. All authors contributed to manuscript revision, read, and approved the submitted version.
